# *GhWRKY6* Acts as a Negative Regulator in Both Transgenic *Arabidopsis* and Cotton During Drought and Salt Stress

**DOI:** 10.3389/fgene.2019.00392

**Published:** 2019-04-26

**Authors:** Zhi Li, Lei Li, Kehai Zhou, Yihao Zhang, Xiao Han, Yanpeng Din, Xiaoyang Ge, Wenqiang Qin, Peng Wang, Fuguang Li, Zhiying Ma, Zhaoen Yang

**Affiliations:** ^1^State Key Laboratory of Cotton Biology (Hebei Base), College of Agronomy, Hebei Agricultural University, Baoding, China; ^2^Institute of Cotton Research, Chinese Academy of Agricultural Sciences, Anyang, China; ^3^Anyang Hospital of Traditional Chinese Medicine, Anyang, China

**Keywords:** *Gossypium hirsutum*, ABA signaling, drought, salt, negative regulation

## Abstract

Drought and high salinity are key limiting factors for cotton production. Therefore, research is increasingly focused on the underlying stress response mechanisms of cotton. We first identified and cloned a novel gene encoding the 525 amino acids in cotton, namely *GhWRKY6*. qRT-PCR analysis indicated that *GhWRKY6* was induced by NaCl, PEG 6000 and ABA. Analyses of germination rate and root length indicated that overexpression of *GhWRKY6* in *Arabidopsis* resulted in hypersensitivity to ABA, NaCl, and PEG 6000. In contrast, the loss-of-function mutant *wrky6* was insensitive and had slightly longer roots than the wild-type did under these treatment conditions. Furthermore, *GhWRKY6* overexpression in *Arabidopsis* modulated salt- and drought-sensitive phenotypes and stomatal aperture by regulating ABA signaling pathways, and reduced plant tolerance to abiotic stress through reactive oxygen species (ROS) enrichment, reduced proline content, and increased electrolytes and malondialdehyde (MDA). The expression levels of a series of ABA-, salt- and drought-related marker genes were altered in overexpression seedlings. Virus-induced gene silencing (VIGS) technology revealed that down-regulation of *GhWRKY6* increased salt tolerance in cotton. These results demonstrate that *GhWRKY6* is a negative regulator of plant responses to abiotic stress via the ABA signaling pathway.

## Introduction

Crops must adjust to various environmental factors including drought, high salinity, and extreme temperatures, which can limit their production and distribution. The plant hormone abscisic acid (ABA) plays diverse roles in plant growth and development, such as seed germination inhibition, dormancy maintenance, stomatal regulation, flowering time, and adaptations to drought, salt and cold stress (Huang et al., 2012; Denance et al., 2013). The ABA-dependent and -independent signal transduction pathways play significant roles in response to osmotic stress in plants (Ishitani et al., 1997). ABA accumulation under stress conditions can stimulate ABA-inducible transcription factors (TFs), such as DREB, bZIP, MYC/MYB, AREB/ABF, NAC, and AP2/ERF (Ciftci-Yilmaz and Mittler, 2008; Dubos et al., 2010; Fujita et al., 2011; Chen et al., 2012; Mizoi et al., 2012; Nakashima et al., 2012; Puranik et al., 2012; Rushton et al., 2012).

The WRKY is one of the largest families of transcriptional regulators in plants (Bencke-Malato et al., 2014). *WRKY* gene products are characterized by the WRKY domain that is composed of 60 amino acids near the N-terminus and a zinc finger structure at the C-terminus (Eulgem et al., 2000; Rushton et al., 2010). Based on the number of WRKY domains and the structure of their zinc fingers, the WRKY family can be divided into three groups (I–III) (Zhang and Wang, 2005; Rushton et al., 2010). The W-box element (TTGACC/T) is the minimal consensus sequence that is required for specific DNA binding, and WRKY preferentially bind to the W-box (Rushton et al., 2010). Furthermore, WRKY are key regulators and play diverse roles in many plant biological processes, including plant responses to pathogens, growth and development, metabolism, morphogenesis of trichomes and embryos, senescence, seed development, biosynthesis, and hormonal signal regulation (Yu et al., 2010; Zhou et al., 2011; Bakshi and Oelmuller, 2014). Increasing studies have focused on the roles of WRKY in abiotic stress (Birkenbihl et al., 2012; Jiang et al., 2012). The *Arabidopsis WRKY63*, *WRKY70*, and *WRKY54* responded to ABA and were found to be important in osmotic stress signaling (Ren et al., 2010; Li et al., 2013). The overexpression of *TaWRKY93* was strongly induced by NaCl and exogenous ABA, and overexpression of *TaWRKY93* in *Arabidopsis* enhanced salt, drought, cold and osmotic stress tolerance (Qin et al., 2015). Overexpression of *VaWRKY14* can increase drought tolerance in *Arabidopsis* by regulating stress-related genes such as *COR15A*, *COR15B*, *COR413*, *KIN2*, and *RD29A* (Zhang et al., 2018). Overexpression of *GhWRKY25* resulted in transgenic tobacco sensitive to drought stress but with enhanced tolerance to salt stress (Liu et al., 2016). *GhWRKY6*-like improved salt tolerance in transgenic *Arabidopsis* through the activation of the ABA signaling pathway and reactive oxygen species (ROS) scavenging (Ullah et al., 2018).

Cotton is one of the most important cash crops worldwide and provides more than 90% of the raw materials for textile fibers (Yang et al., 2018). Abiotic stresses, including drought, high salinity, and low temperature, are major threats to cotton production and have caused significant yield penalties (Matiu et al., 2017; Yang et al., 2017). We cloned and characterized a novel *WRKY* gene member (*GhWRKY6*) from upland cotton. Interference and over accumulation of gene transcription levels showed that *GhWRKY6* may act as a negative regulator of salt-stress response by regulating stress-related genes. Our study not only revealed an important candidate gene for cotton genetic improvement, but also facilitates understanding of salt-stress tolerance mechanisms in cotton.

## Materials and Methods

### Plant Materials and Treatments

Upland cotton accessions ‘ZM9612’ (salt sensitive) and ‘ZM9807’ (salt tolerant) were obtained from the Institute of Cotton Research of the Chinese Academy of Agricultural Sciences. Seeds were sterilized with 3% H_2_O_2_ for 16 h and washed with distilled water. The seeds were subsequently placed on wet filter papers to promote germination at 28°C for 48 h. Uniform seedlings were transferred into pots with nutritional soil and vermiculite (v/v = 1:1) and grown in a greenhouse at 30°C with a 16 h light/8 h dark photoperiod until the third true leaf expanded (at around 21 days old). For salt and drought treatment, the ‘ZM9612’ seedlings were exposed to 400 mM NaCl or 15% polyethylene glycol 6000 (PEG 6000) (w/v) solution. The roots were harvested at 0, 4, 6, 12, 24, and 36 h. For the ABA analysis, 100 μM ABA solution was sprayed onto the ‘ZM9612’ leaves, then the leaves were harvested at 0, 4, 6, 12, 24, and 36 h. The plant tissue samples were immediately frozen in liquid nitrogen and stored at -80°C for RNA extraction.

A *wrky6 Arabidopsis* mutant was obtained from China Agricultural University, as described by Huang et al. (2016). All *Arabidopsis thaliana* plants were grown in pots containing a soil mixture (enriched soil/vermiculite = 1:1) or on solid medium that included MS salts (M519 M&S BASAL, PhytoTechnology Laboratories^TM^, Beijing, China), 2% (w/v) sucrose, and 0.8% (w/v) agar. Seeds were sterilized with 50% (v/v) sodium hypochlorite before they were placed onto the surface of the agar culture medium. Seeds were subsequently vernalised at 4°C for 2 days in the dark before being transferred to an artificial growth chamber (20–21°C, 16 h light/8 h dark photoperiod).

### *GhWRKY6* to W-Box Binding Ability Assay

The Matchmaker Gold Yeast One-Hybrid Library Screening System (Clontech) was used to check *GhWRKY6* binding activities. Three tandem copies of the W-box (TTGACC) or mutant W-box (mW-box, TAGACG) were synthesized by oligonucleotide sequencing and cloned into the pAbAi vector. The pAbAi-W-box or pAbAi-mW-box constructs were transformed into the yeast strain Y1HGold, and different concentrations of aureobasidin A (AbA) were used to test for the background AbA^r^ expression of the reporter strain. The 1575-bp coding sequence of *GhWRKY6* was cloned into the yeast expression vector pGADT7. The pGADT7-*GhWRKY6* and blank pGADT7 constructs were transformed into the yeast strain Y1HGold carrying the pAbAi-W-box or pAbAi-mW-box plasmids following the manufacturer’s protocol (Clontech). Leucine (Leu) and uracil (Ura) deficient synthetic dextrose (SD) medium was used to culture all of the transformed yeast cells.

### *GhWRKY6* Cloning and Ectopic Expression in *Arabidopsis*

The *GhWRKY6* full-length coding sequence was cloned using specific primers (Supplementary Table S1). For overexpression studies, the 35S::*GhWRKY6* vector was constructed by digesting the *GhWRKY6* coding sequence with *Xba I* and *Asc I* (BioLabs). The digested sequence was then inserted into a modified pCAMBIA3300 (Cambia) plant binary vector that contained a Basta resistance gene. This vector was transformed into the *Agrobacterium tumefaciens* strain GV3101 with electric transformation.

We used the floral dip method (Clough and Bent, 1998) to generate transgenic *Arabidopsis* plants and selected positive lines on an MS medium that contained cephalosporin and Basta herbicide. Based on the separation ratio of seedlings grown on the selection medium, lines with segregation ratios of Basta resistant to Basta sensitive (3:1) were used to generate the T_2_ generation, considered to be single-copy insertion lines. The homozygous T_3_ progenies were further confirmed with real-time PCR. The T_3_ generation of transgenic *Arabidopsis* were used for follow-up experiments.

#### Virus-Induced Gene Silencing (VIGS) of *GhWRKY6*

We used the tobacco rattle virus (TRV) system (pTRV-RNA1 and pTRV-RNA2) for VIGS analysis as previously reported by Pang et al. (2013). A 250-bp fragment of *GhWRKY6* was cloned into the pTRV-RNA2 vector to generate TRV::*GhWRKY6* using the *Xba I* and *BamH I* restriction sites. Similarly, we constructed TRV::*GhCLA1* as a visual marker to monitor silencing efficiency. TRV::00 (empty vector) was used as a negative control. All the constructed vectors were introduced into *A. tumefaciens* strain GV3101. We injected the cotyledons of 10-day-old *Gossypium hirsutum* ‘ZM9612’ seedlings as previously reported by Gong et al. (2018), and the seedlings were kept at 25°C. The lines were injected with TRV::*GhCLA1* generated an albino phenotype approximately 2 weeks after infiltration. RT-PCR was used to detect the interference efficiency of *GhWRKY6* using *GhUBQ7* as an internal control.

### RNA Extraction, RT-PCR, and qRT-PCR

Total RNA was isolated using the RNAprep Pure Plant Kit as per the manufacturer’s instructions (Tiangen, Beijing, China). The first strand of cDNA was synthesized with the PrimeScrip^TM^ II 1^st^ Strand cDNA Synthesis Kit (Takara, Dalian, China). For RT-PCR, the following parameters were used: 94°C for 5 min, 27 cycles of 15 s at 98°C, 15 s at 60°C and 30 s at 68°C. The TKS Gflex DNA Polymerase (Takara) was used in RT-PCR. The PCR products were electrophoretically assessed in a 1% agarose gel. qRT-PCR was performed on an ABI 7900 HT system (Applied Biosystems, Foster City, CA, United States) using the SYBR Premix Ex Taq Kit (Takara). The *G. hirsutum UBQ7* and *Arabidopsis thaliana actin2* genes were used as internal controls. All the primers used in this study are listed in Supplementary Table S1.

### Stress Tolerance Assays

For the germination assay, sterilized seeds of overexpression (OE) and wild-type lines were planted on MS medium containing different concentrations of ABA, mannitol, or NaCl, and the germination was recorded for 10 days to calculate the germination rate. To measure root length, seeds were grown in an upright orientation on 1/2 MS medium supplemented with ABA, mannitol, or NaCl. When root length was observed to be different between the lines, we took photographs and measured the root length. Three biological replicates were conducted, and each replicate contained at least ten seedlings. For the salt tolerance assay, OE lines, *wrky6* mutant and wild-type seedlings were grown in soil for 2 weeks, after which they were watered daily with 200 mM NaCl solution for 14 days. For the drought tolerance assay, water was withheld from 3-week-old seedlings for approximately 14 days, after which watering was resumed, and the survival ratio was recorded 7 days later.

### Measurement of Electrolyte Leakage, Malondialdehyde, Proline Content and H_2_O_2_

To detect the degree of damage caused by salt, 2-week-old seedlings were soaked in 200 mM NaCl and leaves were harvested at 0, 6, and 9 h post treatment. Approximately 200 mg of each sample was dried in liquid nitrogen and ground in 2.0 mL tubes using a RETSCH MM 400 Mixer Mill (RETSCH, Germany). Subsequently, a phosphate buffer saline solution (PBS, 0.05 mol/L Tris-HCl, pH 7.4) was added to the 2.0 mL tubes at a ratio of 1:4 (w/v) in an ice water bath. Samples were centrifuged at 4,000 r/min at 4°C for 10–15 min and the supernatant was kept for total protein and malondialdehyde (MDA) content analysis. MDA and proline were, respectively, measured by the MDA and proline assay kits (JianCheng, Nanjing, China) according to manufacturer’s instructions.

For electrolyte leakage analysis, we weighted 200 mg salt-treated leaves, cleaned them with deionized water and dried them with clean filter paper. We subsequently placed the samples into a clean test tube with 10 mL deionized water. To remove air in extracellular spaces, the samples were placed in a vacuum chamber for 10 min before the intake valve of the sample chamber was opened to allow the water to enter these spaces. The test tubes were inverted every 5 min for 30 min at room temperature, after which the relative electrical conductivity was measured by using SC-110 portable conductivity meter (SUNTEX, Shanghai, China). The values of the control and treatment samples were recorded as C1 and C2, respectively, and the test tubes were heated in boiling water for 10 min and cooled at room temperature. The relative electrical conductivity was measured again for both control and treatment samples and recorded as C3 and C4, respectively. Equation 1 was used to evaluate the damage rate. Three biological replicates were performed and the Student *t*-tests was used as the data statistic.

$$\mathrm{Damage}\;\mathrm{rate}\;(\%)\;\;=\;\;\left(1\;-\;\frac{1\;-\;\mathrm{C2/C4}}{1\;-\;\mathrm{C1/C3}}\right)\;\times \;\mathrm{100}\%\;\;\;\;\;\;\;\;\;\;\;\;(1)$$

We used 3,3′-diaminobenzidine (DAB) staining to detect H_2_O_2_ with a DAB chromogenic kit (JianCheng) according to the manufacturer’s instructions.

### Salt Damage Index

We divided the damage degree of cotton under salt stress into five levels: 0, leaves expand and grow normally; I, edges appear dry or yellow, and the whole plant has mild water loss symptoms; II, approximately 50% of the leaves are dry, and the stems of the plants appear dry or yellow; III, more than 80% of the leaf area is necrotic, and more than 50% of the plant stems appear dry; and IV, plants are dead. Equation 2 was used to evaluate the salt damage index:

Salt damage index (%)  =  Σ(Di × Dd)/(Mi × Md) × 100                   (2)

where Di is the number of unhealthy plants; Dd is the grade’s representative value;

Mi is the total number of plants; and Md is the representative value of the highest grade.

### Stomatal Movement and Measurement

Stomatal movement was analyzed as described previously by Li et al. (2014). Briefly, fully expanded rosette leaves were immersed in a buffer containing 20 mM KCl, 1 mM CaCl_2_ and 5 mM MES-KOH (pH 6.15) for 2.5 h in the light at room temperature. The samples were transferred to 8% PEG 6000-PBS buffer for 2.5 h, while the control was treated in PBS buffer. The stomata were imaged by light microscopy at 40× magnification (MicroPublisher^TM^ 5.0 RTV). The width and length of the stomatal aperture were measured from the resulting images using ImageJ v1.37 and analyzed with SPSS Statistics 17.0. Twenty-five stomata were measured for every sample. Three independent biological replicates were performed for each sample.

### mRNA-Seq Analysis

For mRNA-Seq analysis, the third true leaves of TRV::00 and TRV::*GhWRKY6* plants were harvested and immediately frozen in liquid nitrogen. The total RNA was isolated as described above in the “RNA extraction, RT-PCR, and qRT-PCR” section. The sequencing libraries were produced using the TruSeq^TM^ RNA Sample Prep Kit (Illumina, San Diego, CA, United States) according to the manufacturer’s instructions. The HiSeqX-Ten platform was used for sequencing, and the adaptors were removed from the raw RNA-Seq data. TopHat (version 2.0.13) and Cufflinks (version 2.2.1) were used to align and calculate RNA-Seq expression levels, Gene expression was standardized as fragments per kilobase million (FPKM). Cuffdiff (version 2.2.1) was used to identify differentially expressed genes [*q*-value (FDR) ≤ 0.005; absolute value of fold change ≥ 2].

## Results

### Cloning and Verifying the Binding Activity of *GhWRKY6* to W-Box

**Figure 1:**
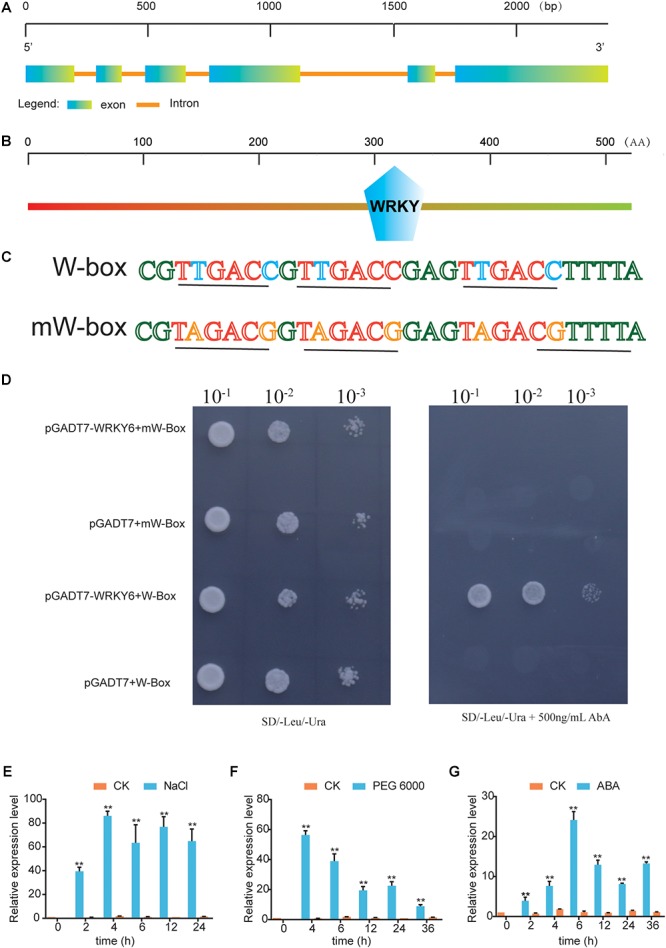
Identification of *GhWRKY6* as a transcriptional regulator and its response to abiotic stress. **(A)** Genomic structure of *GhWRKY6*. **(B)** Conserved WRKY domain in *GhWRKY6*. **(C)** Sequence of the triple tandem repeats of the W-box or mW-box binding elements. **(D)** W-box or mW-box as bait in the yeast one-hybrid system. Yeast cells carrying pGADT7 or pGADT7-*GhWRKY6* were grown on SD/-Leu/-Ura or SD/-Leu/-Ura supplemented with 500 ng/mL AbA. **(E–G)** Expression patterns of *GhWRKY6* in cotton under different abiotic stress conditions. Three-week-old cotton seedlings were subjected to treatment with 100 μM ABA, 400 mM NaCl or 15% PEG 6000. Independent *t*-tests indicated that there were significant differences at ^∗^*p* < 0.05, ^∗∗^*p* < 0.01.

Based on unpublished RNA-Seq data of *G. hirsutum* under NaCl treatment, we identified a novel *WRKY* gene that was significantly upregulated under NaCl treatment. BLASTP and phylogenetic tree analysis showed that this *WRKY* gene had a high degree of sequence similarity with *AtWRKY6*, and it was therefore designated as *GhWRKY6* (Supplementary Figure S3 and Supplementary Table S2). The expression pattern showed that *GhWRKY6* was expressed in vegetative tissues and preferentially expressed in stems (Supplementary Figure S4). We cloned the 1575-bp full-length coding sequence of *GhWRKY6* from the salt-tolerant *G. hirsutum* cultivar ‘ZM9807’. *GhWRKY6* was mapped to chromosome A01 from 11509602 to 11511977 bp and contained six exons (Figure 1A). Protein sequence analysis showed that *GhWRKY6* had one typical WRKY domain (281–341 aa), indicating that it belongs to the WRKY family (Figure 1B).

The functional conservation of the WRKY domain was mirrored by a significant conservation of the W-box binding site (Zhang and Wang, 2005). *WRKY* genes act as both repressors and activators by binding to target gene promoters (Huang et al., 2016). To test whether *GhWRKY6* had W-box binding activity, the yeast one-hybrid system was used to check its DNA-protein interaction. Three repeats of the W-box (TTGACC) or mW-box (TAGACG) were inserted into the pAbAi vector (Figure 1C), which included the AbA^r^ reporter gene. They were than separately integrated into the genome of the yeast strain Y1HGold using the *BstB I* restriction enzyme site. When the prey was absent, 500 ng/mL AbA could suppress the background expression of the ABA^r^ reporter genes in the pAbAi-W-box reporter strain. The yeast clones expressing pAbAi-W-box and pGADT7-*GhWRKY6* grew normally on SD/-Leu/-Ura medium with 500 ng/mL AbA, whereas the strain expressing pGADT7-*GhWRKY6* and pAbAi-mW-box did not, indicating that *GhWRKY6* was able to bind with the W-box element and exhibited general transcriptional activation activity (Figure 1D).

### Expression Patterns of *GhWRKY6* After ABA, NaCl, and Drought Treatment

To comprehensively study the roles of *GhWRKY6* in abiotic stress responses, its expression pattern was evaluated under PEG 6000, ABA, and NaCl treatments in ‘ZM9612’. We found that *GhWRKY6* was significantly up-regulated after drought and salt treatments (Figure 1E,F). After NaCl treatment, *GhWRKY6* displayed an early response that reached peak expression after 4 h; the same was true after PEG 6000 treatment. The plant hormone ABA is essential in mediating plant abiotic stress responses (Yoshida et al., 2014; Zhu, 2016). We found that *GhWRKY6* was up-regulated after ABA treatment (Figure 1G). These results indicated that *GhWRKY6* can respond to abiotic stresses in an ABA-dependent manner.

### Overexpression of *GhWRKY6* in *Arabidopsis* Enhanced ABA Sensitivity During Seed Germination and Root Development

**Figure 2:**
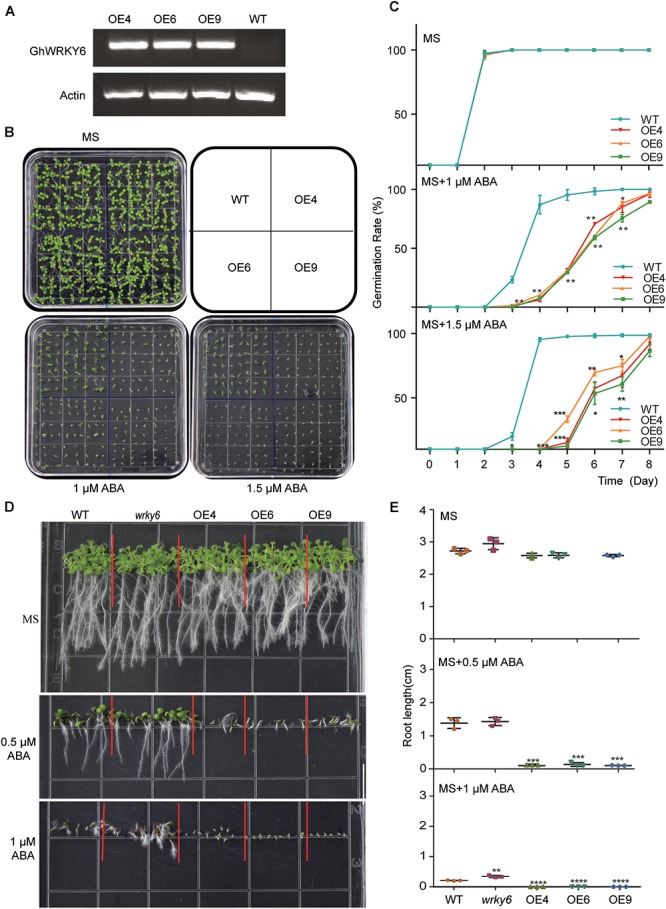
ABA-sensitivity in *GhWRKY6*-overexpressing transgenic *Arabidopsis* lines. **(A)** Expression of *GhWRKY6* in OE lines and wild-type *Arabidopsis* seedlings during seedling development was analyzed by RT-PCR. The *actin2* gene was used as an internal control. **(B)** Phenotypic comparison of seedlings grown for 10 days on MS medium or MS supplemented with 1 and 1.5 μM ABA. **(C)** Seeds were grown on MS or MS medium containing 1 or 1.5 μM ABA. The rates of seed germination were calculated from daily recordings over 10 days of cultivation. **(D,E)** Primary root length was measured in seedlings grown with or without ABA treatment. Seedlings were grown on MS or MS with 0.5 or 1 μM ABA for 10 days, and then photographs were taken so that the length of the roots could be measured. Data are shown as the mean ± SE (*n* = 3). Independent *t*-tests indicated that there were significant differences in both seed germination and root elongation among the wild-type, OE lines and *wrky6* mutant under ABA treatment. ^∗^*p* < 0.05, ^∗∗^*p* < 0.01, ^∗∗∗^*p* < 0.001, ^∗∗∗∗^*p* < 0.0001.

To investigate the function of *GhWRKY6* in plants, OE lines of *GhWRKY6* were generated in *Arabidopsis*, and homozygous T_3_ transgenic lines were used. RT-PCR showed that *GhWRKY6* was well expressed in the three independent transgenic lines, OE4, OE6, and OE9, which were selected for subsequent experiments (Figure 2A). When seedlings were germinated on the MS medium, they grew normally and did not show obvious phenotypic differences (Figure 2B). The germination ratio of wild-type and transgenic seedlings were not different on the MS medium (Figure 2C). However, when these lines were germinated on 1 and 1.5 μM ABA, the OE lines displayed delayed germination initiation, grew more slowly than wild-type seedlings did and showed an ABA sensitive phenotype (Figure 2B,C).

We also tested whether *GhWRKY6* was involved in ABA-mediated root growth. The *wrky6* mutant, OE lines and wild-type seedlings were grown on MS medium with or without ABA. The primary root length was similar between the different genotypes on the MS medium (Figure 2D). However, when these mediums were supplemented with 0.5 μM exogenous ABA, the *GhWRKY6* OE lines seedlings (OE4, OE6, and OE9) had significantly shorter roots than those of wild-type seedlings, but no obvious differences were observed between the *wrky6* mutant and wild-type seedlings. When the concentration of exogenous ABA was increased to 1 μM, the roots of OE line seedlings stopped elongating, whereas the *wrky6* mutant seedlings had longer root length and were less sensitive to ABA than the wild-type seedling (Figure 2D,E). Together, our results indicate that overexpression of *GhWRKY6* in *Arabidopsis* enhanced plant sensitivity to exogenous ABA during germination and root development, and *GhWRKY6* may function as a negative regulator in ABA signaling during germination.

### Overexpression of *GhWRKY6* in *Arabidopsis* Enhanced Sensitivity to Drought and Salt Stress During Seed Germination and Root Development

**Figure 3:**
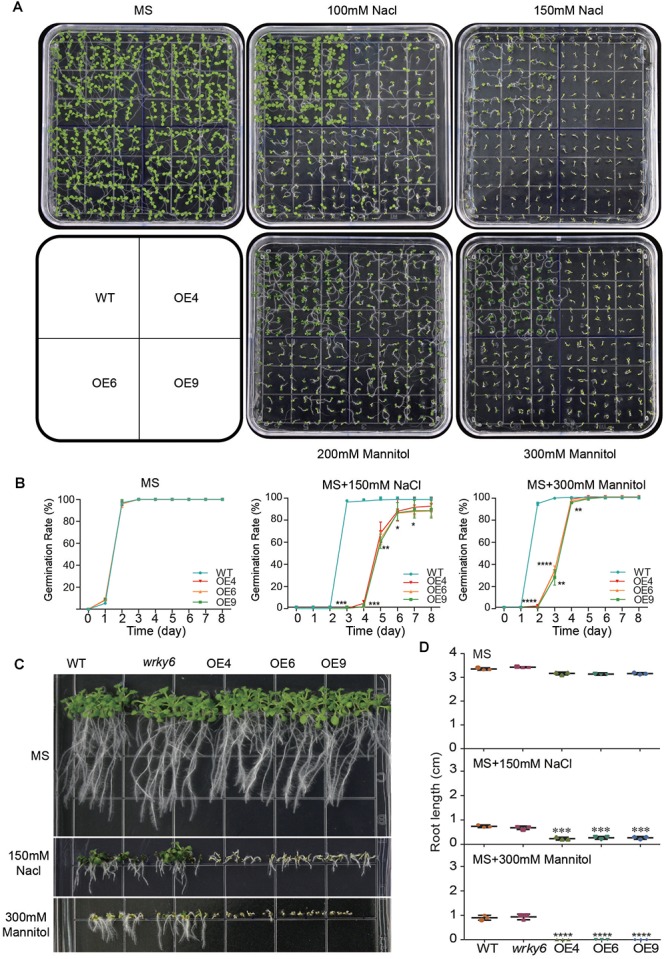
Analysis of germination and root elongation in *GhWRKY6*-overexpressing lines, wild-type and *wrky6* mutant under mannitol and salt stress conditions. **(A)** Phenotypic comparison of seedlings grown on MS medium or MS with 100 mM and 150 mM NaCl, or 200 mM and 300 mM mannitol after 10 days. **(B)** Germination assay of seedlings grown in the conditions described in **(A)**. **(C)** Root elongation of wild-type, OE lines and *wrky6* mutant seedlings after treatment with mannitol and NaCl. **(D)** Statistical analysis of root lengths of seedlings grown in treatment conditions described in **(C)**. Data in **(B,D)** represent the means ± SE from three independent experiments An independent *t*-test indicated that there were significant differences in both seed germination and root elongation among the wild-type, OE lines and *wrky6* mutant under NaCl and mannitol treatments. ^∗^*p* < 0.05, ^∗∗^*p* < 0.01, ^∗∗∗^*p* < 0.001, ^∗∗∗∗^*p* < 0.0001.

To study the roles of *GhWRKY6* during drought or salt stress, OE lines seedlings were exposed to drought or saline conditions. In the MS medium, the wild-type and transgenic seedlings showed uniform growth and germination (Figure 3A,B). However, when seedlings were grown on MS medium supplemented with NaCl or mannitol, the OE lines seedlings (OE4, OE6, and OE9) were sensitive to lower concentrations of NaCl (100 mM) or mannitol (200 mM) compared to wild-type seedlings (Figure 3A). When the concentration of NaCl (150 mM) or mannitol (300 mM) was increased, the cotyledons of OE lines seedlings became bleached and their growth was severely limited (Figure 3A). In addition, the initiation of germination in OE lines seedlings were delayed, and approximately 17% of seeds could not survive in 150 mM NaCl treatment conditions (Figure 3B).

We further measured root length in the *wrky6* mutant, *GhWRKY6*-overexpression lines and wild-type seedlings under salt and drought stresses. The root growth of all genotypes did not displaying differences on the MS medium, but in the presence of 150 mM NaCl or 300 mM mannitol, the root growth in OE lines were severely inhibited, however, the root length of the *wrky6* mutant was similar to that of wild-type (Figure 3C,D). Together, our results show that overexpression of *GhWRKY6* caused greater sensitivity to salt and drought during germination and seedling development in transgenic *Arabidopsis*.

### Transgenic *Arabidopsis* Expressing *GhWRKY6* Displayed Increased ROS Levels and Open Stomata During Drought and Salt Stress

**Figure 4:**
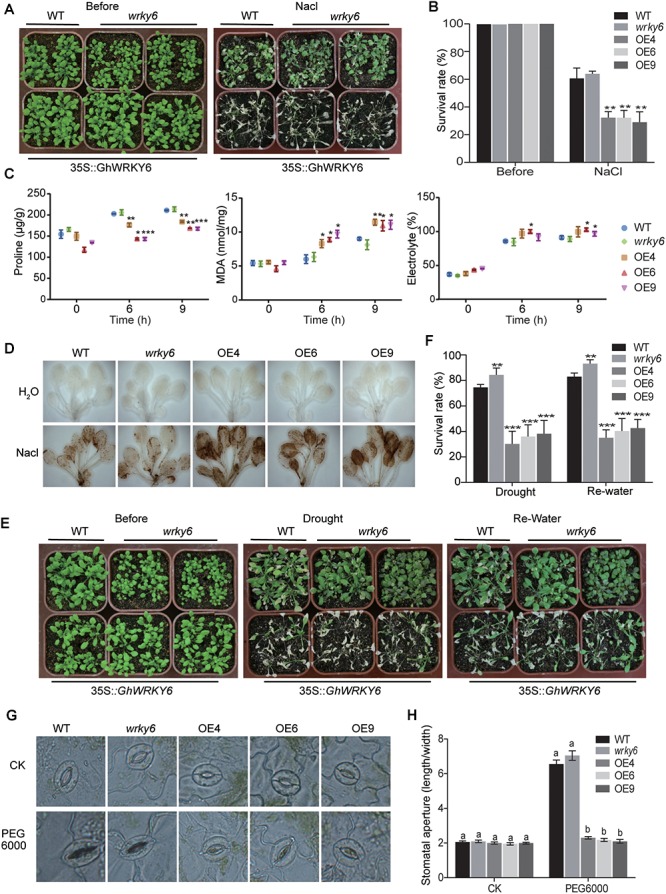
Transgenic *Arabidopsis* expressing *GhWRKY6* display increased ROS levels and open stomata under abiotic stress conditions. **(A)** Sensitivity of wild-type, OE lines and *wrky6* mutant during vegetative growth after 20 days of treatment with 200 mM NaCl. **(B)** Survival rate of transgenic and wild-type *Arabidopsis* grown under saline conditions. **(C)** Proline content, MDA content and electrolyte leakage in wild-type, OE lines and *wrky6* mutant lines during salt stress. **(D)** DAB staining of H_2_O_2_ in *Arabidopsis*. **(E)** Sensitivity of wild-type, OE lines and *wrky6* mutant seedlings to drought stress. Drought stress was imposed by not watering the plants for 20 days, after which watering resumed for 1 week. **(F)** Survival rate of *Arabidopsis* lines after water stress treatment and rehydration. **(G)** Stomatal movement in response to drought treatment in transgenic and wild-type *Arabidopsis*. CK: leaves were placed in PBS until stomata were completely open; PEG 6000: leaves were transferred to 8% PEG 6000 solution for 2.5 h. **(H)** Measurement of stomatal length: width ratio in plants from panel **(G)**. The data represent the means of 25 stomata pooled from three independent experiments. The different letters above the graph bars in **(H)** indicate a significant difference (*p* < 0.05). The data in **(B,C,F)** represent the means ± SE from three independent experiments. Independent *t*-tests indicated that there were significant differences among the wild-type, OE lines and *wrky6* mutant seedlings at ^∗^*p* < 0.05, ^∗∗^*p* < 0.01, ^∗∗∗^*p* < 0.001.

The *wrky6* mutant, wild-type and OE lines seedlings were well watered for 20 days, after which they were watered with 200 mM NaCl for 2 weeks. In the transgenic OE lines (OE4, OE6, and OE9) we observed shrinkage, wilting, chlorosis and even death, whereas the wild-type and *wrky6* mutant seedlings displayed mild symptoms. Moreover, the *wrky6* mutant seedlings showed a slight salt tolerance compared to wild-type seedlings (Figure 4A). Approximately 30% of the OE lines survived compared with the >60% survival rate of the *wrky6* mutant and wild-type seedlings (Figure 4B).

High salinity enhanced the production of ROS and caused ROS-associated injury (Abbasi et al., 2007). To test whether *GhWRKY6* could regulate ROS levels under salt stress, we compared the ROS levels in the OE lines, *wrky6* mutant and wild-type seedlings under normal and saline conditions. Hydrogen peroxide (H_2_O_2_) is a prominent ROS species involved in stress signaling and oxidative injury, We used DAB (3,3′-diaminobenzidine) staining to detect H_2_O_2_. Under normal conditions, H_2_O_2_ accumulated at extremely low levels, and no significant differences were observed among the OE lines, wild-type and *wrky6* mutant seedlings (Figure 4D). When they were exposed to NaCl, all seedlings accumulated H_2_O_2_, as indicated by dark staining. However, the OE lines produced and accumulated more H_2_O_2_ than wild-type and *wrky6* mutant seedlings did. The enrichment of ROS in the OE lines suggested that *GhWRKY6* enhanced ROS-associated oxidative injury.

To further support the DAB staining results, electrolyte leakage and MDA content, which are indictors of membrane injury and membrane lipid peroxidation were examined, respectively (Figure 4C). Under normal growth conditions, there was no difference in the relative electrolyte leakage and MDA content among OE lines, *wrky6* mutant and wild-type seedlings. However, when exposed to salt stress, the relative electrolyte leakage and MDA level in OE lines were significantly higher than those in *wrky6* mutant or wild-type seedlings. The electrolyte leakage level and MDA content in the OE lines was approximately 1.10 and 1.25 times higher than that of wild-type or the *wrky6* mutant seedlings after 9 h of salt stress treatment, respectively. Proline is a stabilizer for proteins and macromolecular complexes, scavenger of free radicals and regulator of cellular redox potential, which play important roles in plant responses to adverse environmental constraints (Ben Rejeb et al., 2012). Under normal conditions, all seedlings had relatively low proline content (Figure 4C), however, salt stress significantly promoted the accumulation of proline. Transgenic OE lines accumulated approximately 18% less proline than wild-type or *wrky6* mutant seedlings did. Our results indicated that *GhWRKY6* plays a negative role in salt stress by accumulating more ROS *in vivo* under salt stress condition.

In the drought stress assay, water was withheld from 3-week-old seedlings for 2 weeks until 50% of the OE lines seedlings were severely wilted (Figure 4E). Watering was then resumed for 1 week, after which the OE lines showed a 30–40% lower recovery rate from the drought symptoms (rolling, shrinkage, chlorosis, delayed growth, or death) compared to the approximately 80 and 90% recovery rates of wild-type and *wrky6* mutant seedlings, respectively (Figure 4F).

The opening and closing of stomata are responsible for gas exchange and water transpiration, and allows plants to respond and adapt to extreme environmental conditions. By controlling the size of the stomatal aperture, the plant can optimizes the efficiency of water use through dynamic changes in the turgor of the guard cells (Daszkowska-Golec and Szarejko, 2013). Under normal conditions, no significant differences were observed in stomatal opening among OE lines, *wrky6* mutant and wild-type seedlings (Figure 4G,H). However, when their leaves were transferred to an 8% PEG 6000 solution, the wild-type and *wrky6* mutant seedlings closed their stomata, while the OE lines remained open stomata (Figure 4G,H). This larger aperture size might reflect more rapid water loss in OE lines than that in wild-type and *wrky6* mutant seedlings under drought stress conditions.

### Overexpression of *GhWRKY6* Altered the Expression of ABA-, Salt-, and Drought-Related Genes

**Figure 5:**
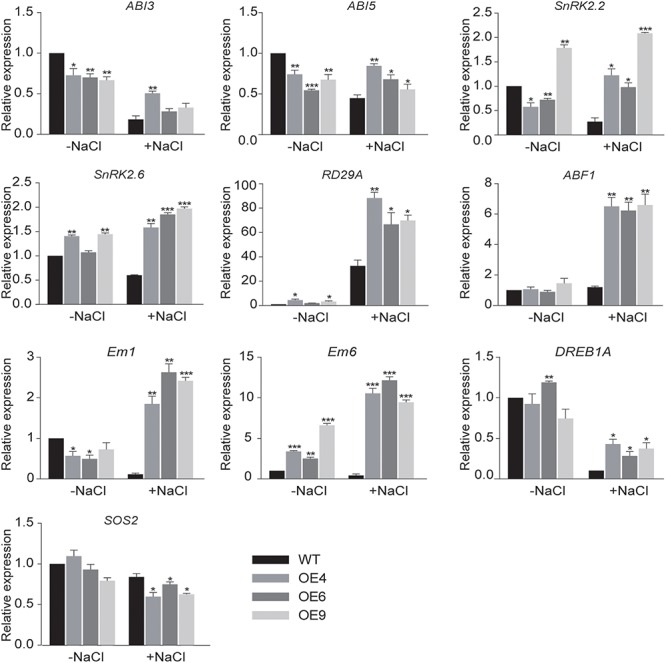
qRT-PCR analysis of expression of stress-related genes in transgenic and wild-type *Arabidopsis*. The 2-week-old wild-type and OE lines seedlings were grown on MS medium and then subjected to 200 mM NaCl treatment condition for 4 h. The seedlings were harvested for qRT-PCR. The values are from three independent experiments. Independent *t*-tests indicated that there were significant differences in the expression of genes among the wild-type and OE lines seedlings at ^∗^*p* < 0.05, ^∗∗^*p* < 0.01, ^∗∗∗^*p* < 0.001.

To better understand the roles of *GhWRKY6* during plant abiotic stress response, the relative expression of marker genes for the ABA signaling pathway, including *ABF1* (Uno et al., 2000), *SnRK2s* (*SnRK2.2* and *SnRK2.6*) (Fujii et al., 2011), Em-like genes (*Em1* and *Em6*) (Gaubier et al., 1993), *ABI3* (Bedi et al., 2016), and *ABI5* (Skubacz et al., 2016) was measured by qRT-PCR in OE lines and wild-type seedlings. The qRT-PCR results showed that transcript levels of ABA-related genes increased in OE lines under salt treatment condition (Figure 5). This indicated that *GhWRKY6* participates in the ABA signaling pathway.

In addition, the expression of the osmotic stress-related genes *SOS2*, *RD29A*, and *DREB1A* was measured in OE lines and wild-type seedlings grown with or without NaCl treatment. The transcription factor DREB1A/CBF3 specifically interacts with the dehydration responsive element DRE/CRT and induced the expression of genes involved in environmental stress tolerance in *Arabidopsis* (Kasuga et al., 2004). In *Arabidopsis*, the *Salt Overly Sensitive 2* (*SOS2*) gene is required for intracellular Na^+^ and K^+^ homeostasis because SOS2 kinase activity is required for salt tolerance (Liu et al., 2000). We found that the relative expression levels of *SOS2* in OE lines were lower than that in wild-type under salt stress, which may be associated with the large stomatal aperture in PEG 6000 treated transgenic *Arabidopsis* and their drought sensitive phenotype. The expression levels of *RD24A* was dramatically affected in the OE lines compared to wild-type under the studied salt stress condition. Cold, drought, and salt can induce *RD29A* gene expression, and the *RD29A* promoter is responsive to drought and cold stress (Msanne et al., 2011). Moreover, *RD29A* is regulated by *ABI3* during germination and vegetative growth (Nakashima et al., 2006). Therefore, our results indicate that *GhWRKY6* might participate in drought and salt stress responses via the ABA signaling pathway by affecting the expression of abiotic stress related genes.

#### VIGS of *GhWRKY6* Enhanced Salt Tolerance in Cotton

**Figure 6:**
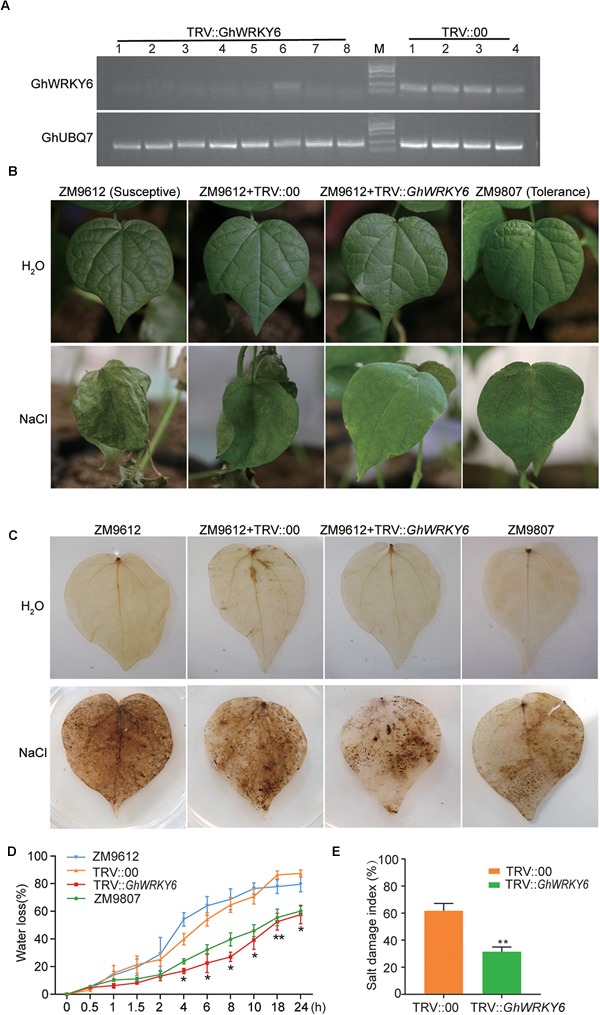
*GhWRKY6*-VIGS cotton plants show enhanced drought sensitivity. **(A)** RT-PCR analysis confirming the efficiency of *GhWRKY6* silencing. **(B)** TRV::*GhWRKY6* plants had more enhanced salt tolerance than TRV::00 control seedlings did. **(C)** DAB staining visualized the accumulation of H_2_O_2_ in cotton plants. **(D)** Leaves harvested from TRV::*GhWRKY6* exhibited less water loss than those from TRV::00 seedlings. **(E)** Salt damage index of TRV::00 and TRV::*GhWRKY6* transgenic lines under saline condition. The data represent the means ± SE from three independent experiments. Independent *t*-tests indicated that there were significant difference in the water loss between the TRV::*GhWRKY6* and TRV::00. ^∗^*p* < 0.05, ^∗∗^*p* < 0.01.

To better understand the function of *GhWRKY6* in cotton, VIGS was used to reduce *GhWRKY6* transcription levels. We used the TRV-based VIGS system to generate *GhWRKY6*-knockdown plants in the salt-sensitive *G. hirsutum* ‘ZM9612,’ and the salt-tolerant *G. hirsutum* ‘ZM9807’ cultivars as a positive control. Twelve days after *Agrobacteria* infiltration of ‘ZM9612,’ the seedlings transformed with TRV::*GhCLA1* displayed an albino phenotype. We used RT-PCR to detect gene expression levels and evaluate silencing efficiency. *GhWRKY6* expression levels were reduced to a larger degree in TRV::*GhWRKY6* plants than in TRV::00 seedling (Figure 6A).

Under normal growth conditions, no stress-related symptoms were observed in TRV::00, TRV::*GhWRKY6* or ‘ZM9612’ and ‘ZM9807’ seedlings. However, when they were treated with 400 mM NaCl for 14 days, the TRV::00 and ‘ZM9612’ seedlings displayed severe stress symptoms, such as leaf shrinkage, yellowing, rolling, wilting, and leaf death. In contrast, the *GhWRKY6* knockdown lines displayed increased salt tolerance and had similar phenotypes to those of the salt tolerant accession ‘ZM9807’ (Figure 6B). According to the symptoms in cotton under salt treatment, we calculated the salt damage index for TRV::00 and TRV::*GhWRKY6* lines, respectively. The salt tolerance of TRV::*GhWRKY6* was significantly improved by nearly onefold compared to that of TRV::00 lines (Figure 6E). Our results support that *GhWRKY6* is a negative regulator of salt tolerance.

The DAB staining showed that the accumulation of H_2_O_2_ was inhibited in the knockdown lines (Figure 6C), which was consistent with the *GhWRKY6* OE lines, indicating that *GhWRKY6* can regulate ROS accumulation under stress conditions. The TRV::*GhWRKY6* seedlings had a lower water loss rate compared to ‘ZM9612’ and TRV::00 seedlings, but was similar to that of the salt-tolerant accession ‘ZM9087’ (Figure 6D), indicating that TRV::*GhWRKY6* had a higher water use efficiency compared to TRV::00 and ‘ZM9612’ seedlings.

### mRNA-Seq Indicated a Potential Mechanism for *GhWRKY6* in Stress Response

**Figure 7:**
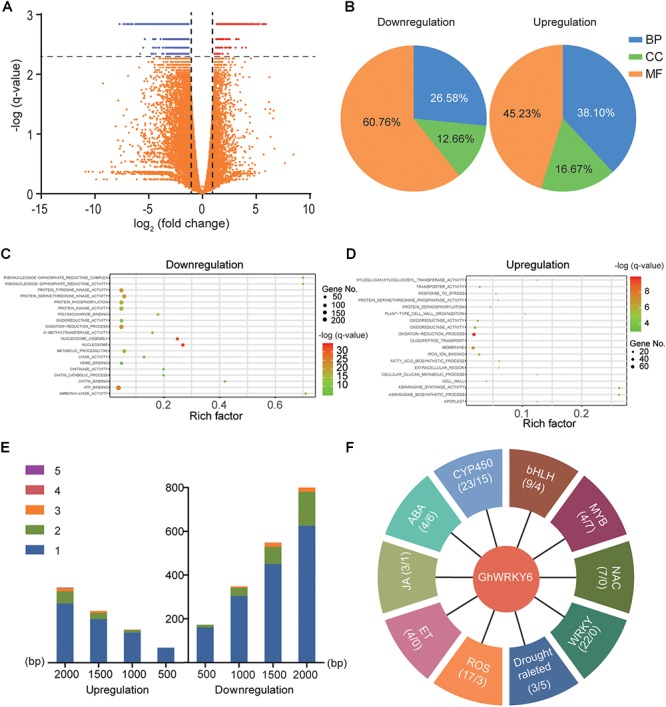
mRNA-Seq analysis for TRV::00 and TRV::*GhWRKY6* seedlings. **(A)** Identification of differentially expressed genes (DEGs) was conducted by adjusted *q*-values (FDR) ≤ 0.005 and the absolute value of fold change ≥ 2. **(B)** All of the DEGs were mapped to GO terms using the agriGO database 2.0. **(C)** Top 20 pathway enrichments in downregulated genes. **(D)** Top 20 pathway enrichments in upregulated genes. **(E)** Scanning for W-boxes in the 2,000-bp region of the promoters of identified DEGs. **(F)** Possible gene families involved in abiotic stress responses.

To further explore the mechanism of *GhWRKY6* in regulating abiotic stress in cotton, RNA-Seq analysis was performed on three independent biological replicates of TRV::00 and TRV::*GhWRKY6* seedlings, respectively. To confirm the reliability of the RNA-Seq data, 16 genes were randomly selected for qRT-PCR analysis. The expression pattern was similar between RNA-Seq and qRT-PCR results (Supplementary Figure S1). The identification of differentially expressed genes (DEGs) was determined by an adjusted *q*-value (FDR) of ≤ 0.005 and an absolute value of fold change of ≥ 2. According to these criteria, a total of 1866 DEGs were identified between TRV::00 and TRV::*GhWRKY6*, including 1372 down-regulated genes and 494 up-regulated genes (Figure 7A).

All DEGs were mapped to GO terms using the agriGO database 2.0 (Tian et al., 2017). In the down-regulated gene cluster, 21 (26.6%), 10 (12.6%), and 48 (60.7%) GO terms were classified as biology process (BP), cellular component (CC) and molecular function (MF), respectively. Similarly, the up-regulated genes included 16 BP (38.1%), 7 CC (16.7%), and 19 MF (45.2%) GO terms (Figure 7B). GO enrichment analysis revealed that the majority of the up-regulated genes belonged to the oxidation-reduction process, membrane, response to stress, and proline biosynthetic process categories (Figure 7D and Supplementary Figure S5). Moreover, the down-regulated genes were enriched in the nucleosome, nucleosome assembly, oxidation-reduction process, and metabolic process categories (Figure 7C and Supplementary Figure S5).

W-box elements (T)TGAC(C/T) are essential binding components that WRKY use to up- or down-regulate target genes. Therefore, we scanned for W-box elements in the 2,000 bp upstream of initiation codons that were potential promoter regions of DEGs. Among the DEGs identified, 735 down-regulated and 346 up-regulated genes contained W-box elements in their promoter regions, occurring in approximately 61 and 70% of the up- or down-regulated genes, respectively. Most of the genes contained one or two W-boxes and the number of genes increased as the scanning region from the initiation codon increased (Figure 7E). According to the literature (Lindemose et al., 2013; Baxter et al., 2014; Verma et al., 2016), the possible gene families involved in stress responses (Figure 7F) include TFs, and genes associated with ROS, plant hormones, and osmotic stress.

Numerous studies have addressed the important roles of TF families in plant defense against abiotic stresses. From the DEGs annotation, we identified group II WRKY *WRKY15*, *WRKY40*, *WRKY11*, and *WRKY18*, group I WRKY *WRKY33* and group III WRKY *WRKY46* (Supplementary Figure S2). The ABA receptor *PYL*, type 2C Protein Phosphatases *PP2Cs*, ABA-Responsive Element (*ABRE*) Binding Factors (*ABFs*) and ABA biosynthesis key enzyme *NCED9* (9-*cis*-epoxycarotenoid dioxygenase) were also identified as DEGs. In addition, genes related to jasmonic acid (JA) and kinetin (ET) have also been identified, including JAZ family genes, *ACS6* (1-aminocyclopropane-1-carboxylate synthase 6), *ACO3* (aconitate hydratase 3), *EBP* (ethylene-responsive TF RAP2-3) and *AOS* (allene oxide synthase). All of these genes respond to osmotic and NaCl stresses (Supplementary Figure S2). Our results suggest that a substantial number of DEGs may be regulated by *GhWRKY6* to form a complex plant stress-tolerant regulatory network.

## Discussion

### *GhWRKY6* Regulates Germination, Early Seedling Development and Stomatal Movement Through ABA Signaling Pathway

WRKY is one of the largest TF families in plants (Rushton et al., 2010). We identified upregulated genes using RNA-Seq datasets from salt treated cotton plants. Bioinformatic prediction and yeast one-hybrid experiments verified a novel gene in the WRKY family, which was designated *GhWRKY6*. When plants were exposed to ABA, high salinity and drought, *GhWRKY6* was significantly up-regulated (Figure 1E–G). When *Arabidopsis* plants were grown on MS medium with supplemented ABA, *GhWRKY6*-overexpressing seedlings exhibited ABA hypersensitivity, whereas *wrky6* mutant lines were insensitive compared to wild-type seedlings (Figure 2B). These results suggest that *GhWRKY6* may play an important role in ABA signaling during seed germination.

*GhWRKY6* in cotton is a homolog of *AtWRKY6* in *Arabidopsis*, indicating a conserved role during seed germination. Moreover, the *AtWRKY6*-overexpressing seedlings presented an ABA hypersensitive phenotype (Huang et al., 2016), which is consistent with our findings in the *GhWRKY6* OE transgenic *Arabidopsis* lines. In *Arabidopsis*, *RAV1* functions upstream and negatively regulates *ABI3*, *ABI4*, and *ABI5*. Biochemical and genetic experiments have verified that *AtWRKY6* can repress *RAV1*, which results in ABA hypersensitivity in *35S::AtWKRY6* expressing lines (Huang et al., 2016). The ABA-response genes *ABI3*, *ABI5*, *SnRK2.2*, *SnRK2.6*, *RD29A*, *ABAF1*, and *DREB1A* transcript levels were elevated in *35S::GhWRKY6* lines (Figure 5). We observed that *GhWRKY6*-overexpressing lines were also hypersensitive to ABA during the early seedling stage, as indicated by their smaller biomass compared to that of wild-type or *wrky6* mutant seedlings. These results are also similar to the *AtWRKY6*-overexpressing lines in *Arabidopsis* and thus suggest that *GhWRKY6* may have conserved roles in ABA hypersensitivity regulation.

In *GhWRKY6*-VIGS cotton lines, the expression level of *ABA2* (Gh_A01G1739) was reduced from approximately 1,500 to less than 10 FPKM. The *ABA2* gene encodes a cytosolic short-chain dehydrogenase involved in the conversion of xanthoxin to ABA-aldehyde during ABA biosynthesis. Four W-boxes were found in the promoter region of *ABA2*, suggesting that *GhWRKY6* could regulate *ABA2* in ABA biosynthesis. It has been reported that ABA inhibits K^+^ and activates anion channels to induce stomatal closure (Wang et al., 1998; Schroeder et al., 2001). Under PEG 6000 treatment, the stomatal closure was less sensitive in *35S::GhWRKY6* than wild-type seedlings, which indicated that *GhWRKY6* regulates stomatal dynamics through ABA signaling pathways. *AtWRKY1* negatively regulates stomatal movement in drought stress via ABA signaling (Qiao et al., 2016), and the overexpression of *GhWRKY25* enhanced sensitivity to mannitol-induced osmotic stress (Liu et al., 2016). With ABA treatment, transgenic plants that overexpressed *GhWRKY17* had larger stomatal apertures than wild-type seedlings did, which might reflect increased water loss rates (Yan et al., 2014).

Increased ABA level is beneficial for plants under stress conditions, because ABA can induce changes at both cellular and whole plant levels (Xiong and Zhu, 2003). The accumulation of ABA is triggered by drought and salt stress. In our mRNA-Seq data, we identified several ABA-response genes that were differentially expressed in the *GhWRKY6*-VIGS lines, including *ABA2*, the ABA receptor *PYL*, type 2C Protein Phosphatases *PP2Cs*, *ABRE*, *ABFs* and the ABA biosynthesis key enzyme *NCED9*, indicating that *GhWRYK6* may regulate stomatal movement by modulating ABA levels *in vivo*, resulting in increased tolerance to abiotic stresses.

In summary, *GhWRKY6* is possibly associated with ABA signaling pathways in cotton and further experimental evidence should be provided to verify its specific roles in abiotic stress responses.

### *GhWRKY6* Plays Important Roles in Abiotic Stress Responses

Expression patterns showed that *GhWRKY6* is expressed in most plant tissues except for the ovule, suggesting that *GhWRKY6* may have diverse roles in the different phases of plant development (Supplementary Figure S4). However, the function of *GhWRKY6* was unclear in cotton. In this study, overexpression of *GhWRKY6* enhanced sensitivity to NaCl and mannitol in *Arabidopsis*. *RAV1* encodes an AP2/B3 domain TF, and *AtWRKY6* can directly bind to the promoter region of *RAV1* in *Arabidopsis* to form an *AtWRKY6-RAV1* regulatory pathway. Previous studies on *Arabidopsis* indicated that this pathway was involved in leaf senescence, flowering, seed germination, and early seedling development. However, the function of *AtWRKY6* in salt and drought stress has not been reported.

Overexpression of *RAVs* resulted in salt- and drought-stress sensitivity phenotypes in *Arabidopsis* (Fu et al., 2014). Compared to the *wrky6* mutant and wild-type lines, the *35S::GhWRKY6* showed enhanced sensitivity to salt and drought conditions, possibly through the binding of *GhWRKY6* to the *RAV1* promoter. Ectopic expression of the cotton *RAV1* gene in *Arabidopsis* also conferred salinity and drought sensitivity to transgenic plants (Li et al., 2015).

Virus-induced gene silencing of *GhWRKY6* can improve the salt tolerance of salt-susceptible cotton, and the mRNA-Seq data of *GhWRKY6*-knockdown lines showed that the cotton *RAV1* (Gh_D13G0717) genes were significantly downregulated (log2 fold change = -2.2 with *q*-value of 0.001) (Supplementary Figure S2). Furthermore, the *RAV1* promoter contained three W-boxes within a 1 Kb region upstream of *RAV1*. Therefore, *GhWRKY6* may directly interact with *RAV1* gene to form the *GhWRKY6*-*RAV1* pathway to regulate salt and drought responses. In addition to the *GhWRKY6*-*RAV1* pathway, other pathways may contribute to the role of *GhWRKY6* in abiotic stress responses. The mRNA-Seq data of *GhWRKY6*-VIGS lines identified a number of DEGs were down-regulated more than twice those that were up-regulated.

The WRKY act primarily on their target genes by binding to the W-box in their promoters. Our experiments revealed that genes for the nucleosome assembly, nucleosome and ribonucleoside-diphosphate reductase complex were enriched in the down-regulated gene cluster, indicating that *GhWRKY6* may regulate transcriptional processes via nucleosome-related genes. GO enrichment revealed that 50 genes were involved in nucleosome and nucleosome assembly, among which 46 had at least one W-box in their promoter region, further indicating that *GhWRKY6* may regulate the expression of nucleosome genes by binding to their promotors.

Approximately 500 genes were up-regulated in *GhWRKY6*-VIGS lines, these genes may be repressed when *GhWRKY6* is present. Genes involved in asparagine biosynthetic processes and asparagine synthase activity were primarily abundant in the up-regulated gene cluster. Accumulation of asparagine in plants can improve osmotic adjustment under saline condition (Renau-Morata et al., 2017). A total of nine asparagine biosynthesis genes were identified at the genome-wide level, of which five were identified as DEGs with W-box in their promoter regions to which *GhWRKY6* could bind directly.

Oxidation-reduction processes had the smallest *q*-value in the GO enrichment, and the gene that encodes an orthologous of ALCOHOL DEHYDROGENASE 1 (*ADH1*; Gh_A01G1605) in *Arabidopsis*, showed a significantly elevated transcription level in *GhWRKY6*-VIGS lines compared to the control. Accumulation of *AtADH1* in *Arabidopsis* enhanced plant tolerance to salt, drought, cold, and pathogen infection (Shi et al., 2017). The 2 Kb region upstream of *GhADH1* contained four W-boxes, suggesting that *ADH1* may be repressed by *GhWRKY6*. Consistent with this hypothesis, *GhADH1* was significantly up-regulated in *GhWRKY6*-VIGS lines, which ultimately resulted in enhanced salt tolerance. The transcript level of lipid transfer protein 3 (*LTP3*, Gh_A10G1332), which responds to salt, drought, cold, and ABA, increased from 26 to 1,000 FPKM (Guo et al., 2013; Pagnussat et al., 2015; Gao et al., 2016). However, the promoter region of *LTP3* contained no W-box, indicating that *GhWRKY6* cannot directly bind to its promoter. Taken together, *GhWRKY6* plays an important role in plant abiotic stress responses by regulating multiple downstream genes (Supplementary Figure S6). It works as a negative regulator in stress response and is a promising candidate for cotton stress improvement via CRISPR-CAS9 genome editing technology.

## Author Contributions

ZY and ZL conceived and designed the study. YD, YZ, WQ, and PW performed the experiments. XH prepared the figures and analyzed the data. XG and ZY wrote part of the manuscript. LL and KZ performed a critical review for intellectual content. FL, ZY, and ZM provided the funding for the experimental project. All authors read, edited, and approved the current version of the manuscript.

## Conflict of Interest Statement

The authors declare that the research was conducted in the absence of any commercial or financial relationships that could be construed as a potential conflict of interest. The reviewer QY declared a past co-authorship with one of the authors FL to the handling Editor.
