# Supplementary material for: GhWRKY6 Acts as a Negative Regulator in Both Transgenic Arabidopsis and Cotton During Drought and Salt Stress
Source: Front Genet. 2019 Apr 26;10:392. doi: 10.3389/fgene.2019.00392 (PMC6497802; doi:10.3389/fgene.2019.00392)
Supplement: Supplementary file 1 [file Data_Sheet_1.PDF]

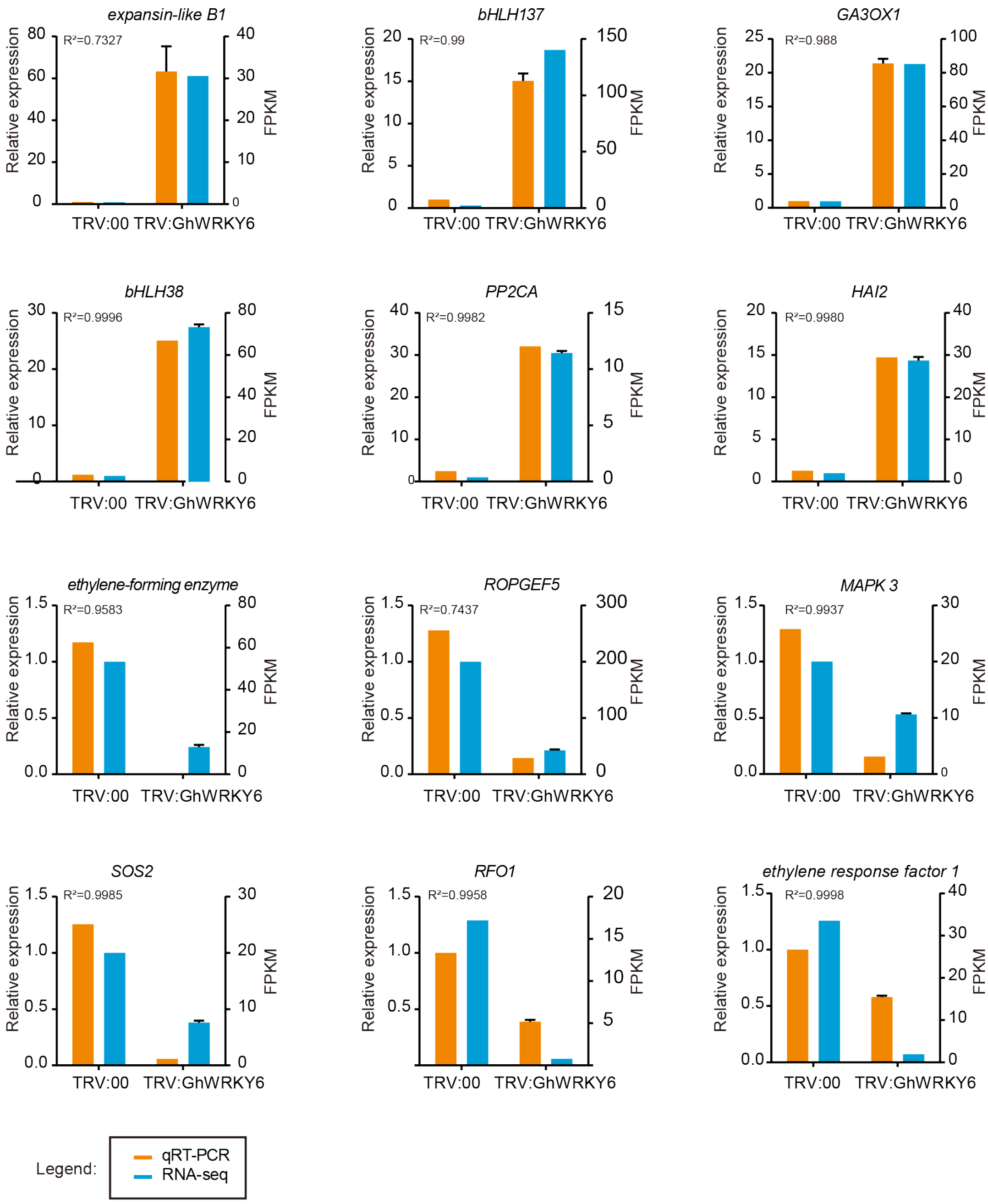

Fig. S1 qRT-PCR validation of transcript levels evaluated by RNA-Seq.

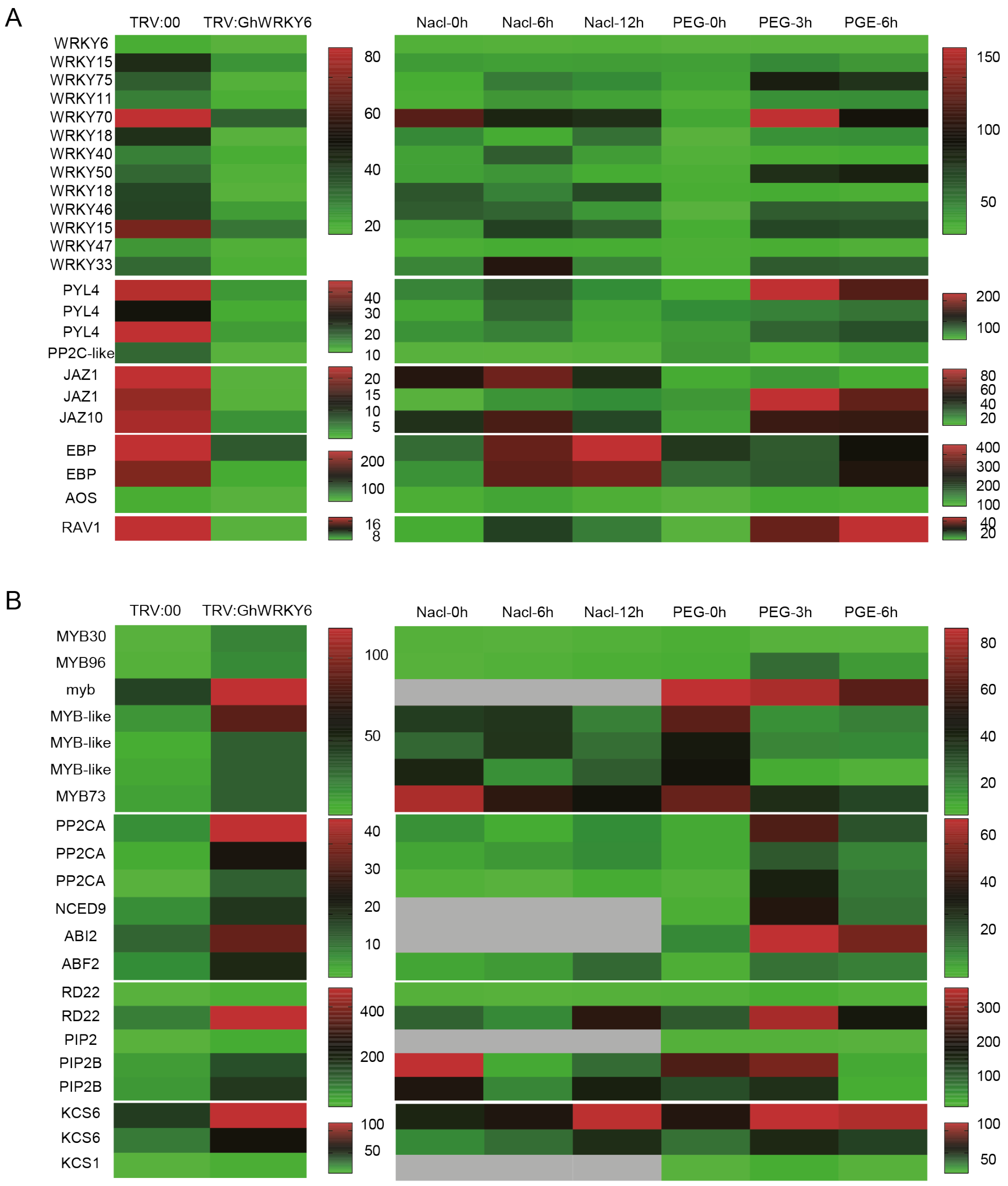

Fig. S2 Part of differential genes and response to abiotic stress.

Table S1. Primer sequences used in this study

Primer Name	Forward Primer(5'-3')	Reverse Primer(5'-3')
GhWRKY6	ATGGAGCCCGTTGATCCTCATATCG	AGTATTGTTGTCTCCGGTGTTTCTT
VIGS-GhWRKY6	GCCATGGCAATGGCATCAAC	CGGCAAACTTGAAATATTGT
Primers for qRT-PCR(Arabidopsis)		
DREB1A	TTACACGGCGGAACAGAGCG	TACGGACGGAAGCGGCAAA
AtRD29A	CAAAGCAATGAGCATGAGCAAG	CGGAAGACACGACAGGAAACAC
ABI3	TCCATTAGACAGCAGTCAAGGTTT	GGTGTCAAAGAACTCGTTGCTATC
ABI5	CACTTCCAGCTCCGCTTTGT	GGTTGTCTAGCCGCAGTCTCA
Em1	TAGGGCACGAGGGTTATCAG	CGCTCTCCACCAGATTTTTC
Em6	GCAAACTCGAAAGGAGCAGT	TCTCGACTCCTTCCTCCTCA
ABF1	TCAACAACTTAGGCGGCGATAC	GCAACCGAAGATGTAGTAGT
SnRK2.2	CCGGAGATCACATCCGATAA	ATGAAATCATCGAGGCAACG
SnRK2.6	AGATCCCGAGGAACCAAAGA	CTCTTTGCAGGGTCAGCAAC
AtSOS2	TTTGGTCTTGCGGGGTTATTC	CGTGTTTTGGGATTGGGGTC
actin2	TCCATGAAACAACTTACAACTCCA	CGTACTCACTCTTTGAAATCCACA
Primers for qRT-PCR(Cotton)		
expansin-like B1	CTGATTTCATCCTCAGCCCCCG	ATCTTGTAACCACCGTAGCGGC
bHLH137	GCTTGAAAGTGGCGAGCAAGTC	ACCTTTTGGTGGCTGTTCGGTT
GA3OX1	ATCAAATGGGCTGGCCCGAAAA	GGTCGGGACAAGCTGGGTAATA
bHLH38	AAACAGAGCACCGACGATGGTT	TGCAGAATTCGAGGTTGTCCCA
PP2CA	CAGATCCCAGCGAAGTCAGTCG	CTAAAACGATGCCGTTCTCCGC
HAI2	AAAATCGTCGACGTGGCTCCAT	CACAAACCGAAGCAACACCGAA
ethylene enzyme	TTGGGGGACACGACGAAAACAA	CATCTGCAGCTGCCACAAACTG
RORGEF5	GCCATTCCCCTACTACAGACGC	GCCCAAGTTCACAGTCCTTGGT
mitogen-activated protein kinase 3	ACTTTGCTGTGCTCGGAGACATT	GGCCGTTGACGATCCAAGAAGT
SOS2	ATACAGGGGTGGAAGTGCCAGA	TGACCTGTTGCAGAGCCAGAAG
RFO1	GCTGTCTTGGTTGCCCCTATGT	TGTGTTCAAACGAAGCCGTCCT
ethylene response factor 1	TCTGGATGGTCATTTGCGCGTT	ATAGTGTCACAACCGATGGCCG
UBQ7	GAAGGCATTCCACCTGACCAAC	CTTGACCTTCTTCTTCTTGTGCTTG
