# Supplementary material for: GhWRKY6 Acts as a Negative Regulator in Both Transgenic Arabidopsis and Cotton During Drought and Salt Stress
Source: Front Genet. 2019 Apr 26;10:392. doi: 10.3389/fgene.2019.00392 (PMC6497802; doi:10.3389/fgene.2019.00392)
Supplement: Supplementary file 2 [file Data_Sheet_2.pdf]

A

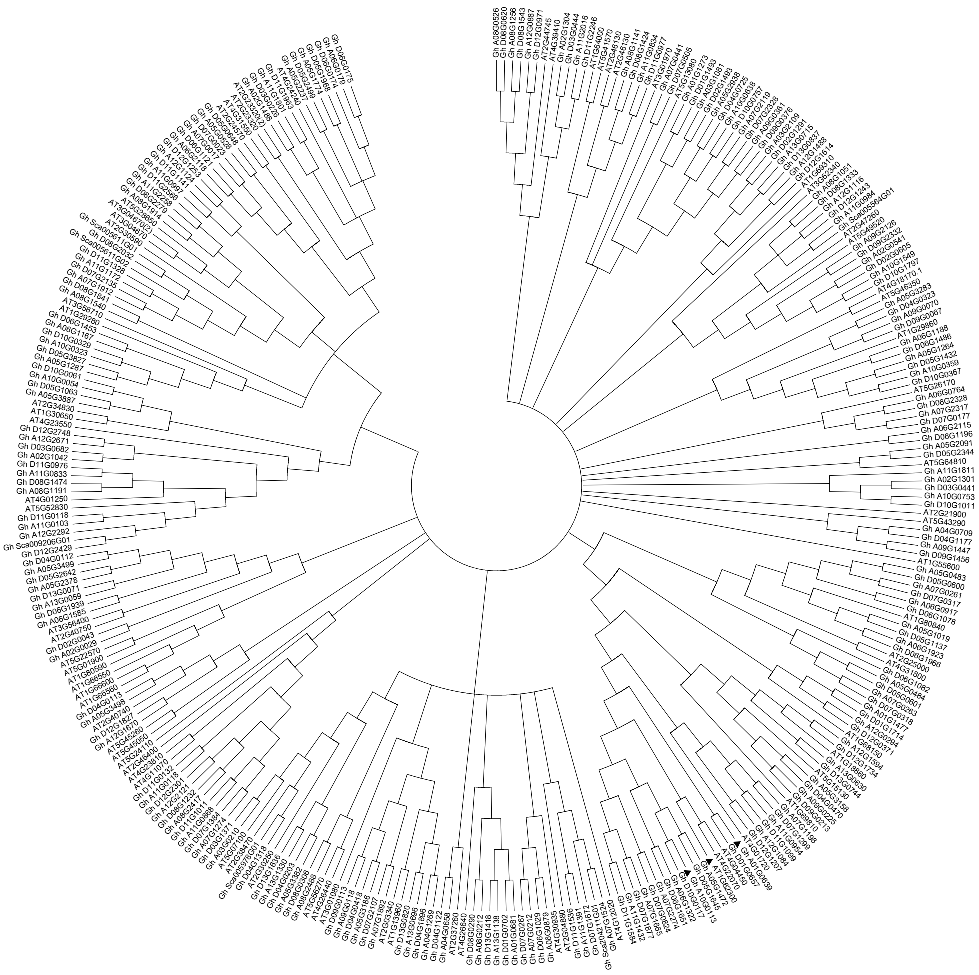

B

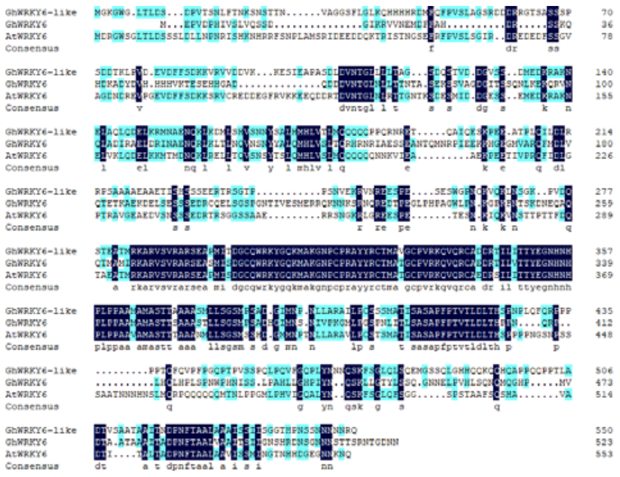

Supplementary Figure 3. Sequence alignment and phylogenetic of Gh_A01G0639 and Gh_A01G0639. (A) Phylogenetic analysis of the WRKY proteins from *Arabidopsis thaliana* and *Gossypium hirsutum*. Gh_A10G0113、Gh_A01G0639 and AT1G62300 are marked with “▲”. (B) Sequence alignment of the *GhWRKY6* protein with AtWRKY6, *GhWRKY6*-like.

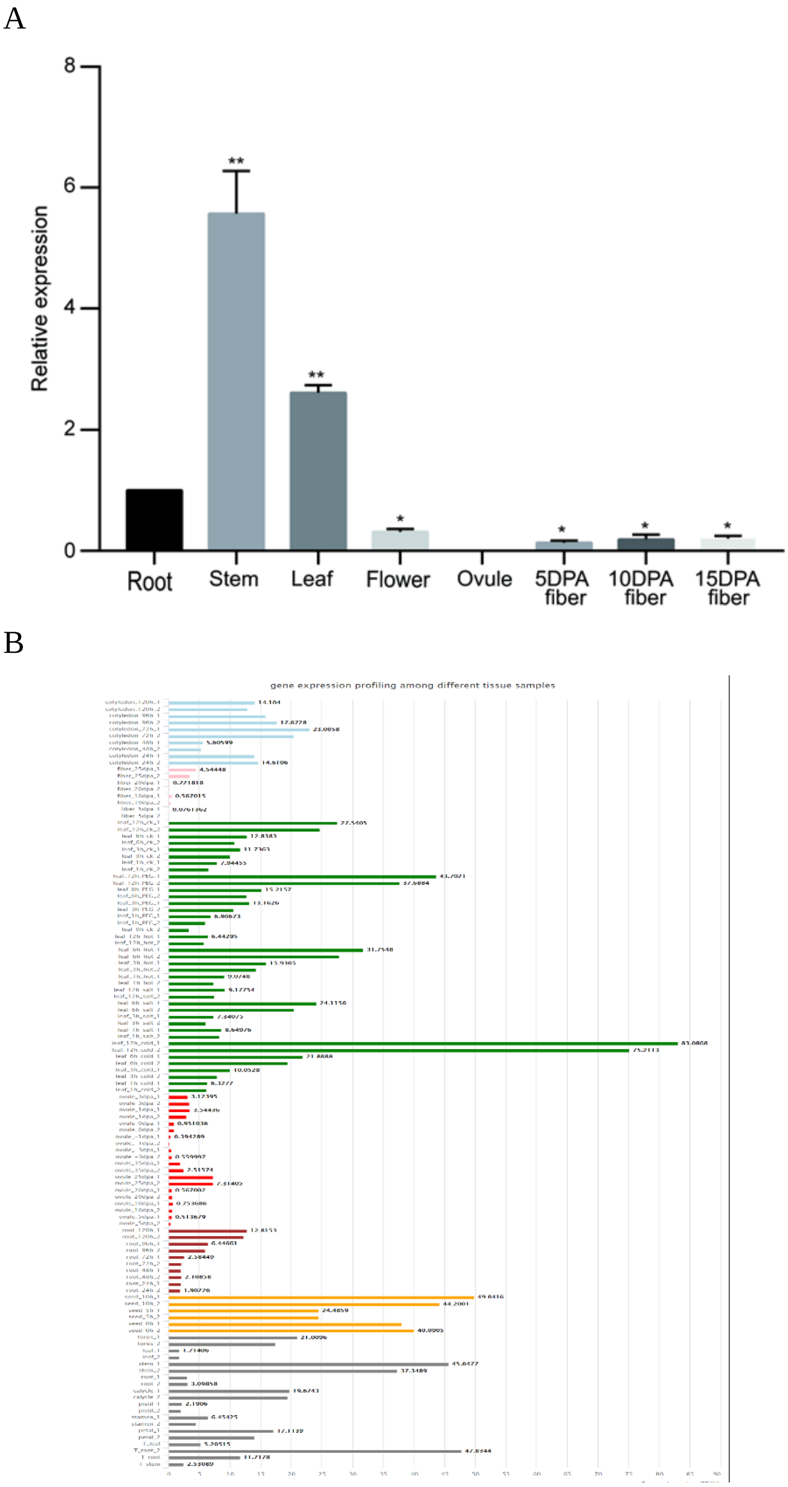

Supplementary Figure 4. (A) Analysis of *GhWRKY6* expression pattern. (B) Analysis of *GhWRKY6*-like expression pattern.

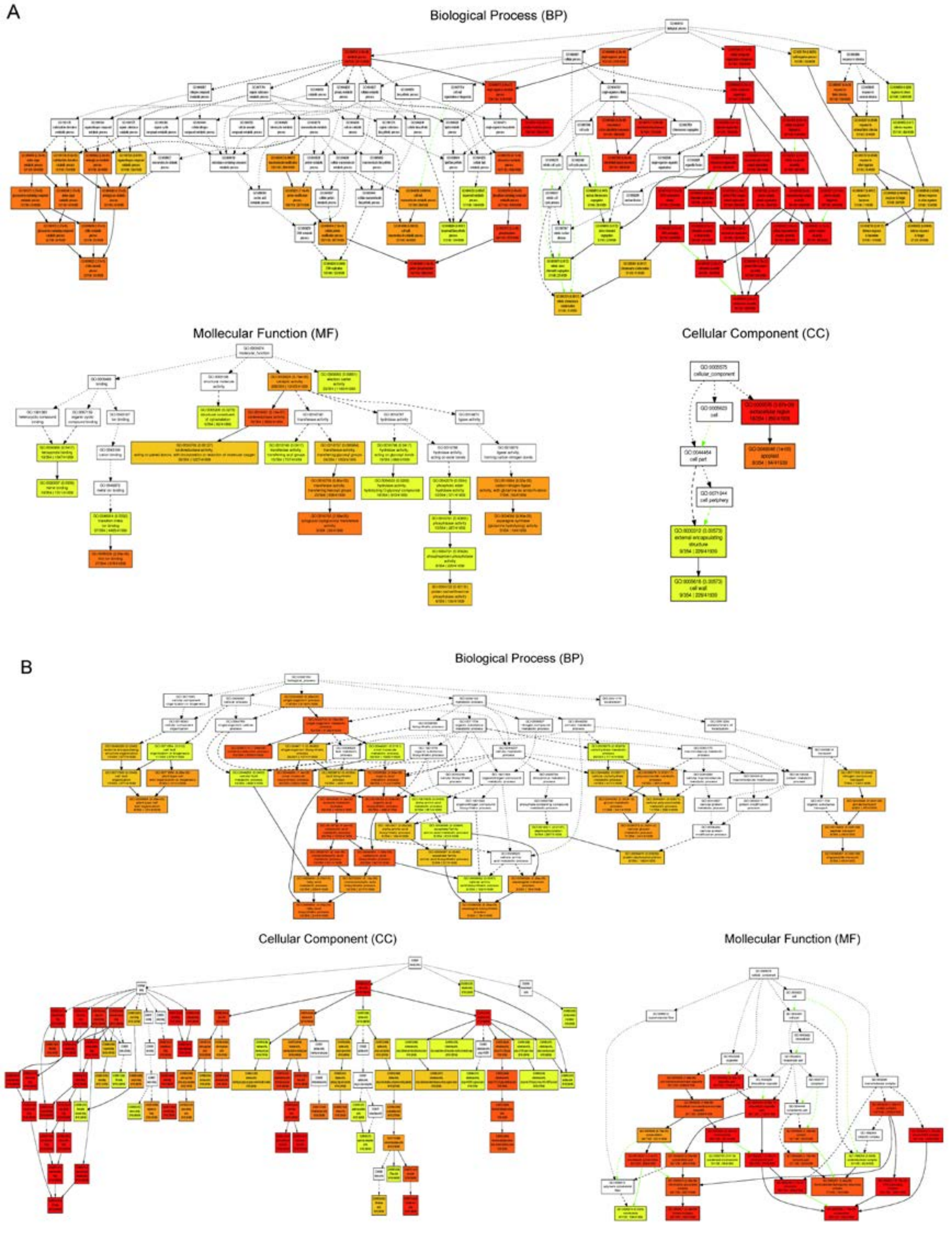

Supplementary Figure 5. The graphical results of GOs. (A) the GOs results of up-regulated DEGs. (B) the GOs results of down-regulated DEGs.

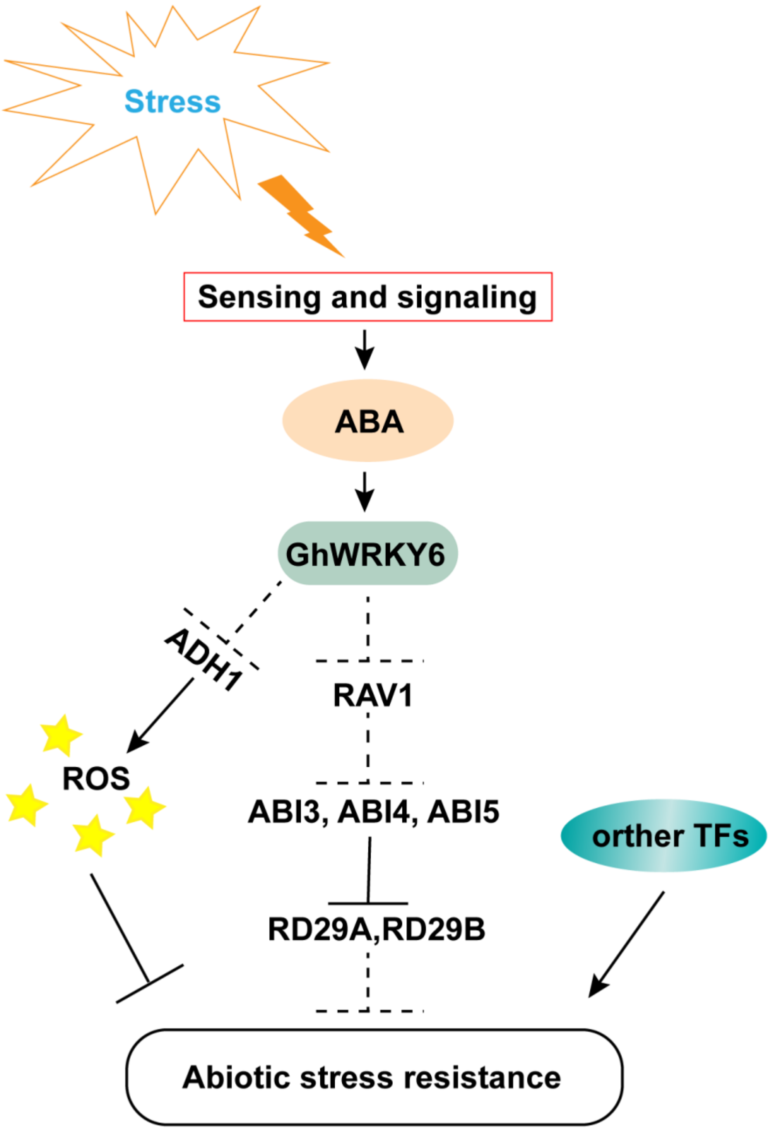

Supplementary Figure 6. Hypothetical model for *GhWRKY6* function.

Supplementary Table 2. *GhWRKY6* align against the Arabidopsis proteins database using BLASTP.

	subject id	Identity (%)	alignment length	E value	bit score
GhWRKY6	AT1G62300.1(AtWRKY6)	46.958	526	1.19E-116	359
GhWRKY6	AT4G04450.1	47.173	513	2.14E-115	355
GhWRKY6	AT4G22070.1	45.81	537	2.06E-95	303
GhWRKY6	AT4G01720.1	38.32	488	4.57E-59	206
GhWRKY6	AT5G15130.1	36.145	332	6.82E-47	173
GhWRKY6	AT1G68150.1	74.49	98	8.59E-46	166
GhWRKY6	AT1G18860.1	61.589	151	6.11E-42	157
GhWRKY6	AT1G69810.1	34.694	343	7.62E-42	155
GhWRKY6	AT4G31800.1	40.26	154	4.94E-25	105
GhWRKY6	AT4G31800.2	40.26	154	4.97E-25	105
GhWRKY6	AT1G80840.1	50.562	89	9.76E-24	102
GhWRKY6	AT2G38470.1	48.889	90	2.69E-23	103
GhWRKY6	AT4G26640.1	44.828	87	7.71E-14	73.9
GhWRKY6	AT4G26640.2	55.128	78	3.78E-23	102
GhWRKY6	AT5G56270.1	36.957	92	4.44E-11	65.1
GhWRKY6	AT1G29860.1	55.128	78	5.32E-23	102
GhWRKY6	AT1G13960.1	36.957	92	4.85E-11	65.1
GhWRKY6	AT4G18170.1	55	80	9.45E-23	102
GhWRKY6	AT1G13960.2	50.82	61	1.08E-11	67.4
GhWRKY6	AT2G25000.1	51.948	77	2.02E-22	97.8
GhWRKY6	AT5G26170.1	55.128	78	2.41E-22	100
GhWRKY6	AT5G49520.1	51.667	60	1.62E-12	69.7
GhWRKY6	AT5G07100.1	53.247	77	2.81E-22	97.8
GhWRKY6	AT5G07100.2	55.128	78	3.08E-22	100
GhWRKY6	AT2G04880.2	51.667	60	2.14E-12	69.3
GhWRKY6	AT2G04880.1	54.054	74	5.04E-22	96.7
GhWRKY6	AT2G47260.1	51.389	72	5.68E-22	94.4
GhWRKY6	AT1G64000.1	50.649	77	7.93E-22	97.8
GhWRKY6	AT5G46350.1	51.282	78	8.03E-22	96.7
GhWRKY6	AT2G03340.1	41.096	73	2.91E-11	64.7
GhWRKY6	AT4G30935.1	51.282	78	8.21E-22	94.7
GhWRKY6	AT2G30250.1	41.096	73	1.37E-11	64.3
GhWRKY6	AT5G41570.1	47.5	80	9.38E-22	98.2
GhWRKY6	AT4G26440.1	34.211	114	3.90E-13	71.6
GhWRKY6	AT3G01080.1	47.5	80	1.22E-21	98.2
GhWRKY6	AT2G46130.1	46.97	66	2.55E-12	68.9
GhWRKY6	AT5G13080.1	43.59	117	1.29E-21	96.3
GhWRKY6	AT2G30590.1	52.381	84	1.46E-21	93.6
GhWRKY6	AT1G69310.2	48.864	88	2.17E-21	95.5
GhWRKY6	AT1G69310.1	52.5	80	3.10E-21	97.1

GhWRKY6	AT1G29280.1	51.667	60	8.59E-13	70.5
GhWRKY6	AT3G62340.1	42.276	123	3.57E-21	96.7
GhWRKY6	AT1G55600.1	51.25	80	4.36E-21	95.5
GhWRKY6	AT2G23320.1	56.944	72	6.24E-21	91.7
GhWRKY6	AT5G28650.1	45.192	104	1.16E-20	95.5
GhWRKY6	AT4G24240.1	47.541	61	5.52E-11	65.1
GhWRKY6	AT1G30650.1	47.561	82	3.75E-20	93.2
GhWRKY6	AT3G58710.1	50	60	9.23E-11	63.9
GhWRKY6	AT3G58710.2	51.316	76	4.37E-20	87.4
GhWRKY6	AT2G44745.1	53.425	73	1.02E-19	87.4
GhWRKY6	AT5G64810.1	41.88	117	1.03E-19	91.3
GhWRKY6	AT3G01970.1	44.828	87	1.64E-19	89.4
GhWRKY6	AT2G34830.1	44.828	87	1.64E-19	89.4
GhWRKY6	AT3G04670.1	51.429	70	3.36E-19	87.8
GhWRKY6	AT4G39410.1	46.067	89	4.47E-19	87.8
GhWRKY6	AT2G24570.1	48.101	79	4.89E-19	90.1
GhWRKY6	AT4G23550.1	57.627	59	5.64E-19	88.2
GhWRKY6	AT4G31550.1	43.182	88	1.19E-18	87
GhWRKY6	AT4G31550.2	50	76	1.40E-18	87.4
GhWRKY6	AT4G01250.1	37.671	146	1.41E-18	88.2
GhWRKY6	AT5G52830.1	52.857	70	1.62E-18	86.3
GhWRKY6	AT2G37260.2	52.857	70	1.65E-18	85.9
GhWRKY6	AT2G37260.1	40.404	99	1.89E-18	85.1
GhWRKY6	AT2G46130.2	43.182	88	1.90E-18	84.7
GhWRKY6	AT5G45050.1	52.778	72	2.79E-18	83.2
GhWRKY6	AT5G45050.2	45.161	93	2.92E-18	87.4
GhWRKY6	AT2G21900.1	42.045	88	5.67E-18	85.1
GhWRKY6	AT5G45260.1	45	80	6.53E-18	84.7
GhWRKY6	AT5G43290.1	48.684	76	8.48E-18	84.3
GhWRKY6	AT2G46400.1	45.679	81	1.90E-17	83.2
GhWRKY6	AT2G40740.1	47.368	76	2.34E-17	83.2
